# Correction: Indirect evidence of sex-selective abortion practices to the imbalanced sex ratio at birth in Australian migrant populations

**DOI:** 10.1371/journal.pgph.0005747

**Published:** 2025-12-30

**Authors:** Amanuel Tesfay Gebremedhin, Gizachew A. Tessema, Ravisha Srinivasjois, Judith A. Daire, Kevin E.K. Chai, Bereket Duko, Kalayu Brhane Mruts, Gavin Pereira

The fifth author’s name is spelled incorrectly. The correct name is: Kevin E.K. Chai. The correct citation is: Gebremedhin AT, Tessema GA, Srinivasjois R, Daire JA, Chai KEK, Duko B, et al. (2025) Indirect evidence of sex-selective abortion practices to the imbalanced sex ratio at birth in Australian migrant populations. PLOS Glob Public Health 5(5): e0004672. https://doi.org/10.1371/journal.pgph.0004672.
