# Supplementary material for: Indirect evidence of sex-selective abortion practices to the imbalanced sex ratio at birth in Australian migrant populations
Source: PLOS Glob Public Health. 2025 May 28;5(5):e0004672. doi: 10.1371/journal.pgph.0004672 (PMC12118887; doi:10.1371/journal.pgph.0004672)
Supplement: S1 File — (DOCX) [file pgph.0004672.s001.docx]

**S1 File. Operational definitions and interpretations**

**The *stopping rule*:** a fertility decision where families chose to have no more additional children after having a desired sex composition of their children (usually after a son). The *stopping rule* can be seen as a form of postnatal sex selection based on the sex outcome of the last child. This rule mainly affects the sex ratio of the last births (SRLB). This mechanism distinguishes its impact from the influence of sex-selective abortion.(9)

**Sex ratio of the last birth (SRLB):**  The sex ratio of the final birth when women have ceased their reproduction (here considered as having their last child at 40-49 years of age). An SRLB higher than the SRB suggests that the *stopping rule* has imbalanced the SRB.(10)

**Parity Stopping Ratio (PSR)** is defined as the proportion of women with a given number of children who stop having children at that parity. In other words, it's the number of women who cease childbearing at a specific parity, divided by the number of women who had a birth at that parity, expressed as a percentage.

**Parity Progression Ratio (PPR):** is the probability that a woman will have a birth at parity i+1, given that she has had a birth at parity i. The PSR is essentially the inverse of the PPR, meaning that PSR = 1 - PPR. So, if you know one, you can calculate the other.

**The sex specific PSR:** The proportion of mothers with a given number of children who stop childbearing (here considered as having their last child at 40-49 years of age) after a male (or female) birth at a specific parity. **PSRm** is the proportion of women who stop childbearing after a male birth at a specific parity and PS**Rf** is the proportion of women who stop childbearing after a female birth at the same parity.This concept was proposed by Pham *et al*.(9) We assume that, in situations where the sex of the child is not a factor in the couples’ fertility decision, the sex specific PSR should be the same, regardless of the sex of the previous birth. So, in societies without son preference, the PSRm and PSRf should be roughly the same, but son preference results in PSRm being greater than PSRf. It should be noted that the ratio of **PSRm/PSRf** is a key indicator of the strength of son preference in a population. A ratio greater than 1 indicates that women are more likely to stop childbearing after a male birth than after a female birth. The higher the ratio, the stronger the influence of son preference is assumed to be.

**Conditional SRB:** The number of males born per 100 females conditioned on previous sex for second and higher-order births. Here we computed the conditional SRB using a sub-cohort of mothers with two consecutive births (*parity 0-1 cohort,*) and three consecutive births (*parity 0-1-2 cohort).*

For the *parity 0-1 cohort,* we presented the SRB for each group (parity 0: firstborn; parity 1: second born; M: second birth, firstborn was male; F: second birth, firstborn was female).

For the *parity 0-1-2 cohort,* we presented the SRB for the following groups (parity 2: third born: no conditioning; MM: third birth, first and second birth were male; FF: Third birth, first and second births were female; Mixed: one male, one female in any order).

**SI References**

1. Australian Bureau of Statistics (1990) Australian Standard Classification of Countries for Social Statistics (ASCSS) 1990. (ABS; 1990 [cited 2023 January 24]. Available from: <https://www.abs.gov.au/AUSSTATS/abs@.nsf/DetailsPage/1269.01990?OpenDocument>, Canberra).

2. Australian Bureau of Statistics (1998) Standard Australian Classification of Countries (SACC) 1998. (ABS; 1998 [cited 2023 January 24]. Available from: <https://www.abs.gov.au/AUSSTATS/abs@.nsf/allprimarymainfeatures/C72FCA469016EB0ACA25726800189A43?opendocument>, Canberra).

3. Australian Bureau of Statistics (2008) Standard Australian Classification of Countries 2^nd^ edition (SACC 2^nd^ edition). (ABS; 2008 [cited 2023 January 24]. Available from: <https://www.abs.gov.au/AUSSTATS/abs@.nsf/Lookup/1269.0Main+Features1Second%20Edition?OpenDocument>, Canberra).

4. Australian Bureau of Statistics (2011) Standard Australian Classification of Countries 2011. (ABS; 2011 [cited 2023 January 24]. Available from: <https://www.abs.gov.au/AUSSTATS/abs@.nsf/allprimarymainfeatures/F63BD8670E4CF23CCA257FDC0011DB93?opendocument>, Canberra).

5. K. Chowdhary, Natural language processing. *Fundamentals of artificial intelligence*, 603-649 (2020).

6. P. M. Nadkarni, L. Ohno-Machado, W. W. Chapman, Natural language processing: an introduction. *Journal of the American Medical Informatics Association* **18**, 544-551 (2011).

7. K. Edvardsson, M.-A. Davey, R. Powell, A. Axmon, Sex ratios at birth in Australia according to mother’s country of birth: A national study of all 5 614 847 reported live births 1997–2016. *PloS one* **16**, e0251588 (2021).

8. B. N. Pham, W. Hall, P. S. Hill, Indirect evidence of the contribution of prenatal sex selection practices to the high sex ratio at birth in Vietnam. *Journal of Population Research* **28**, 293-299 (2011).

9. B. N. Pham, T. Adair, P. S. Hill, C. Rao, The impact of the stopping rule on sex ratio of last births in Vietnam. *Journal of Biosocial Science* **44**, 181-196 (2012).

10. G. Dalla Zuanna, T. Leone, A gender preference measure: the sex-ratio at last birth. *Genus*, 33-56 (2001).
