# Supplementary figures and images for: Indirect evidence of sex-selective abortion practices to the imbalanced sex ratio at birth in Australian migrant populations
Source: PLOS Glob Public Health. 2025 May 28;5(5):e0004672. doi: 10.1371/journal.pgph.0004672 (PMC12118887; doi:10.1371/journal.pgph.0004672)

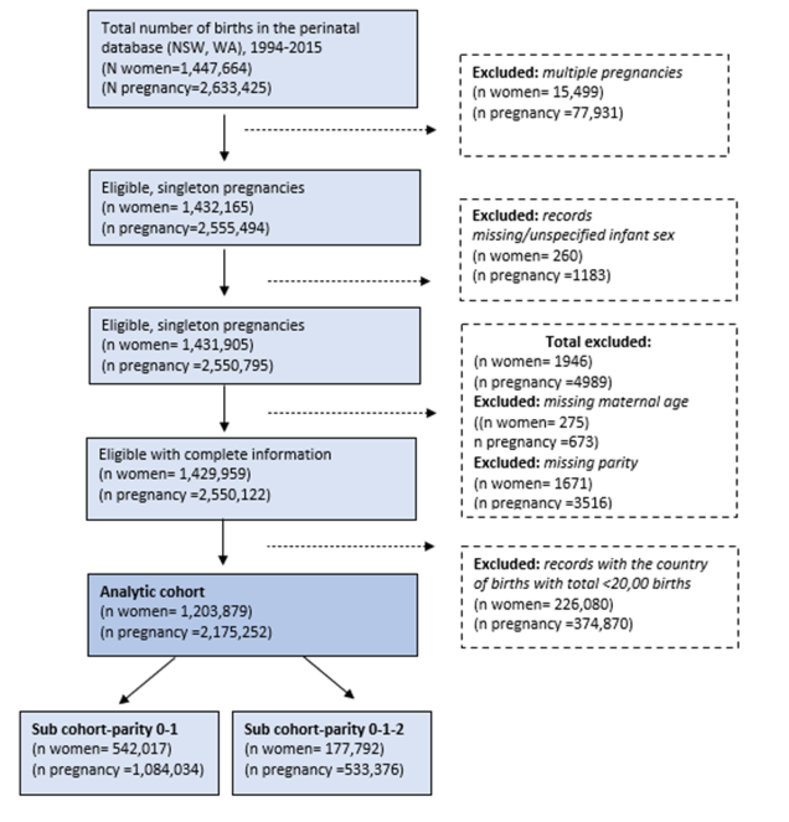

Supplement: S1 Fig — (TIF) [file pgph.0004672.s002.tif]

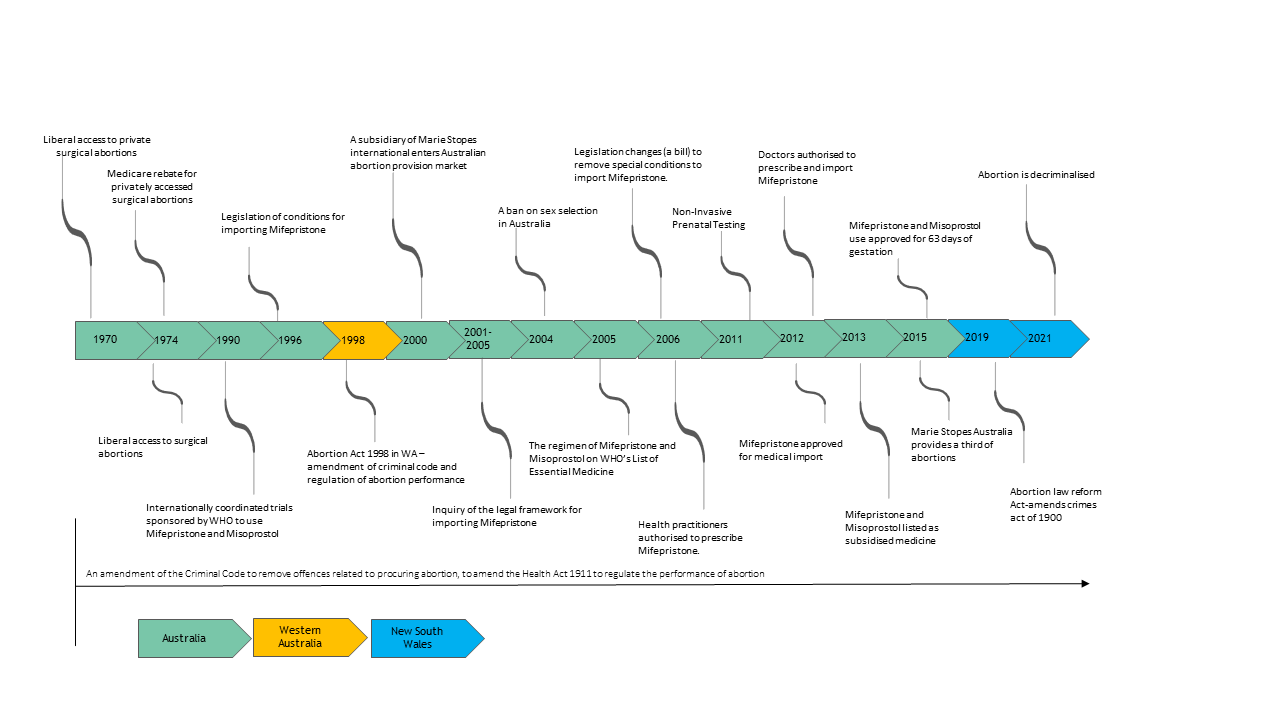

Supplement: S2 Fig — (TIF) [file pgph.0004672.s003.tif]
