# Supplementary material for: Indirect evidence of sex-selective abortion practices to the imbalanced sex ratio at birth in Australian migrant populations
Source: PLOS Glob Public Health. 2025 May 28;5(5):e0004672. doi: 10.1371/journal.pgph.0004672 (PMC12118887; doi:10.1371/journal.pgph.0004672)
Supplement: S1 Table — (DOCX) [file pgph.0004672.s004.docx]

| **S1 Table. Male-to-female ratios of singleton births in Australia (WA, NSW) from 1994 to 2015 by mother's country of birth and stratified by sex of previous sibling(s) for mothers with their first two (or three) consecutive births** | | | | | |
| --- | --- | --- | --- | --- | --- |
| **Country** | **Parity** | **Sex of the previous sibling** | **No of births** | **No of males/Females** | **Sex Ratio (95% CI)** |
| **Australia** |  |  |  |  |  |
|  | **0** | **-** | 456941 | 236100/220841 | 1.069 (1.063,1.075) |
|  | **1** | **-** | 456854 | 234359/222495 | 1.053 (1.047,1.059) |
|  |  | **M** | 236097 | 121116/114981 | 1.053 (1.045,1.062) |
|  |  | **F** | 220757 | 113243/107514 | 1.054 (1.045,1.062) |
|  | **2** | **-** | 156876 | 80735/76124 | 1.061 (1.050,1.071) |
|  |  | **MM** | 46291 | 23835/22456 | 1.061 (1.042,1.081) |
|  |  | **FF** | 39383 | 20176/19207 | 1.050 (1.030,1.071) |
|  |  | **Mixed** | 71185 | 36724/34461 | 1.066 (1.050,1.081) |
| **China** |  |  |  |  |  |
|  | **0** | **-** | 14887 | 7569/7318 | 1.034 (1.002,1.068) |
|  | **1** | **-** | 14895 | 7771/7124 | 1.091 (1.056,1.126) |
|  |  | **M** | 7579 | 3926/3653 | 1.075 (1.027,1.124) |
|  |  | **F** | 7316 | 3845/3471 | 1.108 (1.058,1.160) |
|  | **2** | **-** | 1920 | 1011/909 | 1.112 (1.017,1.216) |
|  |  | **MM** | 515 | 252/263 | 0.958 (0.806,1.139) |
|  |  | **FF** | 615 | 352/263 | 1.338 (1.141,1.570) |
|  |  | **Mixed** | 790 | 407/383 | 1.061 (0.924,1.222) |
| **India** |  |  |  |  |  |
|  | **0** | **-** | 9154 | 4501/4653 | 0.967 (0.928,1.008) |
|  | **1** | **-** | 9243 | 4845/4398 | 1.102 (1.058,1.148) |
|  |  | **M** | 4549 | 2340/2209 | 1.059 (0.999,1.122) |
|  |  | **F** | 4694 | 2505/2189 | 1.144 (1.081,1.212) |
|  | **2** | **-** | 985 | 522/463 | 1.127 (0.995,1.278) |
|  |  | **MM** | 242 | 118/124 | 0.952 (0.740,1.224) |
|  |  | **FF** | 326 | 185/141 | 1.312 (1.054,1.633) |
|  |  | **Mixed** | 417 | 219/198 | 1.106 (0.913,1.340) |
| **New Zealand** |  |  |  |  |  |
|  | **0** | **-** | 13600 | 7054/6546 | 1.078 (1.042,1.114) |
|  | **1** | **-** | 13671 | 6978/6693 | 1.043 (1.008,1.078) |
|  |  | **M** | 7098 | 3600/3498 | 1.029 (0.982,1.078) |
|  |  | **F** | 6573 | 3378/3195 | 1.057 (1.007,1.110) |
|  | **2** | **-** | 4213 | 2135/2078 | 1.027 (0.967,1.091) |
|  |  | **MM** | 1183 | 582/601 | 0.968 (0.864,1.085) |
|  |  | **FF** | 1079 | 542/537 | 1.009 (0.896,1.137) |
|  |  | **Mixed** | 1951 | 1011/940 | 1.076 (0.984,1.175) |
| **UK** |  |  |  |  |  |
|  | **0** | **-** | 24245 | 12467/11778 | 1.058 (1.032,1.085) |
|  | **1** | **-** | 24178 | 12331/11847 | 1.041 (1.015,1.067) |
|  |  | **M** | 12360 | 6333/6027 | 1.051 (1.014,1.088) |
|  |  | **F** | 11818 | 5998/5820 | 1.030 (0.994,1.068) |
|  | **2** | **-** | 5728 | 2939/2789 | 1.054 (1.001,1.110) |
|  |  | **MM** | 1720 | 879/841 | 1.045 (0.951,1.149) |
|  |  | **FF** | 1490 | 796/694 | 1.147 (1.036,1.270) |
|  |  | **Mixed** | 2518 | 1264/1254 | 1.008 (0.932,1.090) |
| **Vietnam** |  |  |  |  |  |
|  | **0** | **-** | 10364 | 5286/5078 | 1.041 (1.002,1.082) |
|  | **1** | **-** | 10364 | 5286/5078 | 1.033 (0.994,1.074) |
|  |  | **M** | 5266 | 2662/2604 | 1.015 (0.962,1.072) |
|  |  | **F** | 5096 | 2604/2473 | 1.053 (0.997,1.113) |
|  | **2** | **-** | 2558 | 1359/1199 | 1.133 (1.049,1.225) |
|  |  | **MM** | 704 | 382/322 | 1.186 (1.023,1.376) |
|  |  | **FF** | 754 | 386/368 | 1.049 (0.909,1.210) |
|  |  | **Mixed** | 1100 | 591/509 | 1.161 (1.031,1.307) |
| **Lebanon** |  |  |  |  |  |
|  | **0** | **-** | 6615 | 3928/3233 | 1.049 (1.000,1.101) |
|  | **1** | **-** | 6603 | 3436/3137 | 1.085 (1.034,1.139) |
|  |  | **M** | 3394 | 1714/1680 | 1.020 (0.954,1.091) |
|  |  | **F** | 3209 | 1722/1487 | 1.158 (1.080,1.241) |
|  | **2** | **-** | 3840 | 1936/1904 | 1.017 (0.954,1.083) |
|  |  | **MM** | 1019 | 510/509 | 1.002 (0.886,1.133) |
|  |  | **FF** | 939 | 465/474 | 0.981 (0.863,1.115) |
|  |  | **Mixed** | 1882 | 961/921 | 1.043 (0.953,1.142) |
| **Philippines** |  |  |  |  |  |
|  | **0** | **-** | 6201 | 3259/2942 | 1.108 (1.054,1.164) |
|  | **1** | **-** | 6205 | 3242/2963 | 1.094 (1.041,1.150) |
|  |  | **M** | 3263 | 1692/1571 | 1.077 (1.006,1.154) |
|  |  | **F** | 2942 | 1550/1392 | 1.114 (1.036,1.197) |
|  | **2** | **-** | 1689 | 888/801 | 1.109 (1.008,1.220) |
|  |  | **MM** | 514 | 280/234 | 1.197 (1.006,1.423) |
|  |  | **FF** | 388 | 202/186 | 1.086 (0.890,1.325) |
|  |  | **Mixed** | 787 | 406/381 | 1.066 (0.927,1.226) |

0: Parity 0 (firstborn); 1: Parity 1 (second born); 2: Parity 2 (Third born); M: Second birth first born was male; F: Second birth first born was female; MM: Two previous males FF: Two previous females; Mixed: One male and one female
