# Supplementary material for: Indirect evidence of sex-selective abortion practices to the imbalanced sex ratio at birth in Australian migrant populations
Source: PLOS Glob Public Health. 2025 May 28;5(5):e0004672. doi: 10.1371/journal.pgph.0004672 (PMC12118887; doi:10.1371/journal.pgph.0004672)
Supplement: S2 Table — (DOCX) [file pgph.0004672.s005.docx]

| **S2 Table. Quantifying the role of stopping rule and sex-selective abortion using sex-specific parity stopping ratio (PSR) in Australia (WA, NSW) from 1994 to 2015 by mother's country of birth** | | | | | | | | | | | | |
| --- | --- | --- | --- | --- | --- | --- | --- | --- | --- | --- | --- | --- |
| **Country, parity** | **All births** |  |  | **Last births** |  |  |  | **Sex-specific PSR** | |  | **Components** |  |
|  | **Males** | **Females** | **Total births** | **Males** | **Females** | **Total** | **Actual SRLB** |  |  | **Total** | **SRB** | $PS{R_{m}^{i}}/PSR_{f}^{i}$ |
|  | **A** | **B** |  | **C** | **D** |  |  | **E** | **F** |  | **G** | **H** |
| *Formula* |  |  |  |  |  |  |  | *C/A*100*  $PSR_{m}^{i}$ | *D/B*100* |  | *A/B*100* | *E/F* |
| **Australia** |  |  |  |  |  |  |  |  | $PSR_{f}^{i}$ |  |  |  |
| 0 | 9,163 | 8,315 | 17,478 | 3,375 | 3,177 | 6,552 | 106.2 | 36.8 | 38.2 | 37.5 | 110.2 | 0.96 |
| 1 | 7,579 | 6,990 | 14,569 | 4,364 | 4,134 | 8,498 | 105.6 | 57.6 | 59.1 | 58.3 | 108.4 | 0.97 |
| 2 | 4,594 | 4,436 | 9,030 | 2,794 | 2,756 | 5,550 | 101.4 | 60.8 | 62.1 | 61.5 | 103.6 | 0.98 |
| 3+ | 6,149 | 5,980 | 12,129 | 3,537 | 3,495 | 7,032 | 101.2 | 57.5 | 58.4 | 58.0 | 102.8 | 0.98 |
|  | 27,485 | 25,721 | 53,206 | 14,070 | 13,562 | 27,632 | 103.7 | 51.2 | 52.7 | 51.9 | 106.9 | 0.97 |
| **China** |  |  |  |  |  |  |  |  |  |  |  |  |
| 0 | 609 | 555 | 1,164 | 323 | 269 | 592 | 120.1 | 53.0 | 48.5 | 50.9 | 109.7 | 1.09 |
| 1 | 730 | 684 | 1,414 | 560 | 492 | 1,052 | 113.8 | 76.7 | 71.9 | 74.4 | 106.7 | 1.07 |
| 2 | 284 | 238 | 522 | 241 | 177 | 418 | 136.2 | 84.9 | 74.4 | 80.1 | 119.3 | 1.14 |
| 3+ | 91 | 75 | 166 | 79 | 62 | 141 | 127.4 | 86.8 | 82.7 | 84.9 | 121.3 | 1.05 |
|  | 1,714 | 1,552 | 3,266 | 1,203 | 1,000 | 2,203 | 120.3 | 70.2 | 64.4 | 67.5 | 110.4 | 1.09 |
| **India** |  |  |  |  |  |  |  |  |  |  |  |  |
| 0 | 153 | 148 | 301 | 71 | 86 | 157 | 82.6 | 46.4 | 58.1 | 52.2 | 103.4 | 0.80 |
| 1 | 130 | 122 | 252 | 99 | 88 | 187 | 112.5 | 76.2 | 72.1 | 74.2 | 106.6 | 1.06 |
| 2 | 70 | 70 | 140 | 55 | 53 | 108 | 103.8 | 78.6 | 75.7 | 77.1 | 100.0 | 1.04 |
| 3+ | 58 | 39 | 97 | 42 | 30 | 72 | 140.0 | 72.4 | 76.9 | 74.2 | 148.7 | 0.94 |
|  | 411 | 379 | 790 | 267 | 257 | 524 | 103.9 | 65.0 | 67.8 | 66.3 | 108.4 | 0.96 |
| **New Zealand** |  |  |  |  |  |  |  |  |  |  |  |  |
| 0 | 438 | 385 | 823 | 180 | 167 | 347 | 107.8 | 41.1 | 43.4 | 42.2 | 113.8 | 0.95 |
| 1 | 354 | 364 | 718 | 229 | 241 | 470 | 95.0 | 64.7 | 66.2 | 65.5 | 97.3 | 0.98 |
| 2 | 233 | 200 | 433 | 154 | 129 | 283 | 119.4 | 66.1 | 64.5 | 65.4 | 116.5 | 1.02 |
| 3+ | 402 | 372 | 774 | 236 | 212 | 448 | 111.3 | 58.7 | 57.0 | 57.9 | 108.1 | 1.03 |
|  | 1,42 | 1,321 | 2,748 | 799 | 749 | 1,548 | 106.7 | 56.0 | 56.7 | 56.3 | 108.0 | 0.99 |
| **UK** |  |  |  |  |  |  |  |  |  |  |  |  |
| 0 | 1,186 | 1,073 | 2,259 | 478 | 432 | 910 | 110.6 | 40.3 | 40.3 | 40.3 | 110.5 | 1.00 |
| 1 | 939 | 954 | 1,893 | 617 | 657 | 1,274 | 93.9 | 65.7 | 68.9 | 67.3 | 98.4 | 0.95 |
| 2 | 558 | 498 | 1,056 | 392 | 362 | 754 | 108.3 | 70.3 | 72.7 | 71.4 | 112.0 | 0.97 |
| 3+ | 554 | 553 | 1,107 | 378 | 374 | 752 | 101.1 | 68.2 | 67.6 | 67.9 | 100.2 | 1.01 |
|  | 3,237 | 3,078 | 6,315 | 1,865 | 1,825 | 3,690 | 102.2 | 57.6 | 59.3 | 58.4 | 105.2 | 0.97 |
| **Vietnam** |  |  |  |  |  |  |  |  |  |  |  |  |
| 0 | 284 | 255 | 539 | 113 | 101 | 214 | 111.9 | 39.8 | 39.6 | 39.7 | 111.4 | 1.00 |
| 1 | 262 | 274 | 536 | 161 | 150 | 311 | 107.3 | 61.5 | 54.7 | 58.0 | 95.6 | 1.12 |
| 2 | 161 | 179 | 340 | 116 | 129 | 245 | 89.9 | 72.0 | 72.1 | 72.1 | 89.9 | 1.00 |
| 3+ | 135 | 129 | 264 | 110 | 95 | 205 | 115.8 | 81.5 | 73.6 | 77.7 | 104.7 | 1.11 |
|  | 842 | 837 | 1,679 | 500 | 475 | 975 | 105.3 | 59.4 | 56.8 | 58.1 | 100.6 | 1.05 |
| **Lebanon** |  |  |  |  |  |  |  |  |  |  |  |  |
| 0 | 185 | 164 | 349 | 55 | 47 | 102 | 117.0 | 29.7 | 28.7 | 29.2 | 112.8 | 1.04 |
| 1 | 174 | 144 | 318 | 62 | 43 | 105 | 144.2 | 35.6 | 29.9 | 33.0 | 120.8 | 1.19 |
| 2 | 135 | 165 | 300 | 49 | 53 | 102 | 92.5 | 36.3 | 32.1 | 34.0 | 81.8 | 1.13 |
| 3+ | 445 | 410 | 855 | 232 | 213 | 445 | 108.9 | 52.1 | 52.0 | 52.0 | 108.5 | 1.00 |
|  | 939 | 883 | 1,822 | 398 | 356 | 754 | 111.8 | 42.4 | 40.3 | 41.4 | 106.3 | 1.05 |
| **Philippines** |  |  |  |  |  |  |  |  |  |  |  |  |
| 0 | 257 | 223 | 480 | 117 | 111 | 228 | 105.4 | 45.5 | 49.8 | 47.5 | 115.2 | 0.91 |
| 1 | 285 | 237 | 522 | 186 | 134 | 320 | 138.8 | 65.3 | 56.5 | 61.3 | 120.3 | 1.15 |
| 2 | 206 | 188 | 394 | 156 | 132 | 288 | 118.2 | 75.7 | 70.2 | 73.1 | 109.6 | 1.08 |
| 3+ | 180 | 188 | 368 | 135 | 141 | 276 | 95.7 | 75.0 | 75.0 | 75.0 | 95.7 | 1.00 |
|  | 928 | 836 | 1,764 | 594 | 518 | 1,112 | 114.7 | 64.0 | 62.0 | 63.0 | 111.0 | 1.03 |

SRB: Sex Ratio at Birth; SRLB: Sex Ratio at Last Birth; PSR: Parity Stopping Ratio; $PSR_{m}^{i}$ Parity Stopping Ratio of Males; $PSR_{f}^{i}$ Parity Stopping Ratio of Females; UK: United Kingdom
