# Supplementary material for: Indirect evidence of sex-selective abortion practices to the imbalanced sex ratio at birth in Australian migrant populations
Source: PLOS Glob Public Health. 2025 May 28;5(5):e0004672. doi: 10.1371/journal.pgph.0004672 (PMC12118887; doi:10.1371/journal.pgph.0004672)
Supplement: S3 Table — (DOCX) [file pgph.0004672.s006.docx]

| **S3 Table. Male-to-female ratios of singleton births in Australia (WA, NSW) by mother's country of birth and stratified by sex of previous births, 1994-2015.** | | | | | | | |
| --- | --- | --- | --- | --- | --- | --- | --- |
| **Country** | **Sex of the previous sibling** | **No of births** | | **No of males/Females** | | **Sex Ratio (95% CI)** | |
| **Australia** |  |  | |  | |  | |
|  | No previous birth | 248806 | 127387/121419 | | 1.049 (1.041,1.057) | |  |
|  | >1st birth: no previous males | 366746 | 191705/175041 | | 1.095 (1.088,1.102) | |  |
|  | >1st birth: one previous male | 262156 | 129976/132180 | | 0.983 (0.976,0.991) | |  |
|  | >1st birth: +1 previous male | 72596 | 35914/36682 | | 0.979 (0.965,0.993) | |  |
| **China** |  |  |  | |  | |  |
|  | No previous birth | 21015 | 11021/9994 | | 1.103 (1.073,1.133) | |  |
|  | >1st birth: no previous males | 17532 | 9469/8063 | | 1.174 (1.140,1.210) | |  |
|  | >1st birth: one previous male | 8503 | 4366/4137 | | 1.055 (1.011,1.101) | |  |
|  | >1st birth: +1 previous male | 716 | 353/363 | | 0.975 (0.842,1.129) | |  |
| **India** |  |  |  | |  | |  |
|  | No previous birth | 16656 | 8711/7945 | | 1.096 (1.064,1.130) | |  |
|  | >1st birth: no previous males | 13566 | 7337/6229 | | 1.178 (1.139,1.218) | |  |
|  | >1st birth: one previous male | 5186 | 2664/2522 | | 1.056 (1.000,1.115) | |  |
|  | >1st birth: +1 previous male | 403 | 208/195 | | 1.067 (0.877,1.297) | |  |
| **New Zealand** | | | | | | |  |
|  | No previous birth | 12164 | 6197/5967 | | 1.039 (1.002,1.076) | |  |
|  | >1st birth: no previous males | 17153 | 8886/8267 | | 1.075 (1.043,1.108) | |  |
|  | >1st birth: one previous male | 9050 | 4557/4493 | | 1.014 (0.973,1.057) | |  |
|  | >1st birth: +1 previous male | 2259 | 1148/1111 | | 1.034 (0.952,1.123) | |  |
| **UK** |  |  |  | |  | |  |
|  | No previous birth | 17380 | 8780/8600 | | 1.021 (0.991,1.052) | |  |
|  | >1st birth: no previous males | 28325 | 14629/13696 | | 1.068 (1.044,1.093) | |  |
|  | >1st birth: one previous male | 14729 | 7286/7443 | | 0.979 (0.948,1.011) | |  |
|  | >1st birth: +1 previous male | 2571 | 1278/1293 | | 0.988 (0.915,1.068) | |  |
| **Vietnam** |  |  |  | |  | |  |
|  | No previous birth | 7015 | 3728/3287 | | 1.134 (1.082,1.189) | |  |
|  | >1st birth: no previous males | 9966 | 5369/4597 | | 1.168 (1.123,1.215) | |  |
|  | >1st birth: one previous male | 6119 | 3053/3066 | | 0.996 (0.947,1.047) | |  |
|  | >1st birth: +1 previous male | 1170 | 595/575 | | 1.035 (0.923,1.160) | |  |
| **Lebanon** |  |  |  | |  | |  |
|  | No previous birth | 2197 | 1128/1069 | | 1.055 (0.971,1.147) | |  |
|  | >1st birth: no previous males | 6758 | 3721/3037 | | 1.225 (1.168,1.285) | |  |
|  | >1st birth: one previous male | 4601 | 2378/2223 | | 1.070 (1.010,1.133) | |  |
|  | >1st birth: +1 previous male | 2191 | 1139/1052 | | 1.083 (0.996,1.177) | |  |
| **Philippines** |  |  |  | |  | |  |
|  | No previous birth | 6818 | 3428/3390 | | 1.011 (0.964,1.060) | |  |
|  | >1st birth: no previous males | 9412 | 4899/4513 | | 1.086 (1.043,1.130) | |  |
|  | >1st birth: one previous male | 4181 | 2107/2074 | | 1.016 (0.956,1.079) | |  |
|  | >1st birth: +1 previous male | 874 | 464/410 | | 1.132 (0.991,1.292) | |  |
