# Supplementary material for: Indirect evidence of sex-selective abortion practices to the imbalanced sex ratio at birth in Australian migrant populations
Source: PLOS Glob Public Health. 2025 May 28;5(5):e0004672. doi: 10.1371/journal.pgph.0004672 (PMC12118887; doi:10.1371/journal.pgph.0004672)
Supplement: S4 Table — (DOCX) [file pgph.0004672.s007.docx]

**S4 Table. Number and percentage of hospital separations with induced abortion stratified by gestational age and year in WA, 2009-2015**

| Maternal Country of birth | Year | Induced abortion admission^1^ | | | | |
| --- | --- | --- | --- | --- | --- | --- |
|  |  |  | **Gestational age (completed weeks)^2^, No (%)** | | | |
|  |  | **No (%)** | **<=13 weeks** | **14-19 weeks** | **>20 weeks** | **Unspecified or not reported** |
| Australia |  |  |  |  |  |  |
|  | **2009** | 2482 (3.55) | 1830 (73.82) | 550 (22.19) | 70 (2.82) | 29 (1.17) |
|  | **2010** | 2691 (3.8) | 1834 (68.15) | 763 (28.35) | 66 (2.45) | 28 (1.04) |
|  | **2011** | 2526 (3.46) | 2310 (91.45) | 131 (5.19) | 54 (2.14) | 31 (1.23) |
|  | **2012** | 2661 (3.56) | 2416 (90.79) | 155 (5.82) | 59 (2.22) | 31 (1.16) |
|  | **2013** | 2682 (3.66) | 2389 (89.08) | 149 (5.56) | 71 (2.65) | 73 (2.72) |
|  | **2014** | 2519 (3.49) | 2290 (90.91) | 122 (4.84) | 65 (2.58) | 42 (1.67) |
|  | **2015** | 2347 (3.31) | 2121 (90.37) | 111 (4.73) | 62 (2.64) | 53 (2.26) |
|  | **2009-2015** | 17908 (3.55) | 15190 (84.84) | 1981 (11.06) | 447 (2.5) | 287 (1.6) |
| India |  |  |  |  |  |  |
|  | **2009** | 25 (4) | 12 (48) | 12 (48) | sup | 0 (0) |
|  | **2010** | 56 (5.86) | 36 (64.29) | 16 (28.57) | 4 (7.14) | 0 (0) |
|  | **2011** | 85 (6.27) | 81 (95.29) | sup | sup | 0 (0) |
|  | **2012** | 110 (7.42) | 106 (96.36) | 0 (0) | sup | sup |
|  | **2013** | 135 (6.81) | 128 (94.81) | sup | sup | 0 (0) |
|  | **2014** | 174 (7.57) | 171 (98.28) | sup | 0 (0) | sup |
|  | **2015** | 177 (6.58) | 173 (97.74) | sup | sup | sup |
|  | **2009-2015** | 762 (6.69) | 707 (92.78) | 35 (4.59) | 15 (1.97) | 5 (0.66) |
| China |  |  |  |  |  |  |
|  | **2009** | 21 (4.41) | 6 (28.57) | 10 (47.62) | sup | sup |
|  | **2010** | 18 (2.84) | 9 (50) | 9 (50) | 0 (0) | 0 (0) |
|  | **2011** | 31 (3.72) | 30 (96.77) | 0 (0) | 0 (0) | sup |
|  | **2012** | 39 (3.53) | 34 (87.18) | sup | 0 (0) | 0 (0) |
|  | **2013** | 55 (4.89) | 50 (90.91) | sup | sup | 0 (0) |
|  | **2014** | 69 (5.1) | 64 (92.75) | sup | sup | sup |
|  | **2015** | 64 (4.77) | 56 (87.5) | sup | sup | 0 (0) |
|  | **2009-2015** | 297 (4.33) | 249 (83.84) | 31 (10.44) | 12 (4.04) | 5 (1.68) |
| Others |  |  |  |  |  |  |
|  | **2009** | 468 (2.15) | 272 (58.12) | 169 (36.11) | 19 (4.06) | sup |
|  | **2010** | 557 (2.39) | 316 (56.73) | 210 (37.7) | 27 (4.85) | sup |
|  | **2011** | 586 (2.33) | 510 (87.03) | 42 (7.17) | 31 (5.29) | sup |
|  | **2012** | 656 (2.4) | 597 (91.01) | 24 (3.66) | 25 (3.81) | 10 (1.52) |
|  | **2013** | 624 (2.25) | 556 (89.1) | 42 (6.73) | 16 (2.56) | 10 (1.60) |
|  | **2014** | 715 (2.5) | 639 (89.37) | 40 (5.59) | 28 (3.92) | sup |
|  | **2015** | 695 (2.34) | 601 (86.47) | 36 (5.18) | 46 (6.62) | 12 (1.73) |
|  | **2009-2015** | 4301 (2.34) | 3491 (81.17) | 563 (13.09) | 192 (4.46) | 55 (1.28) |
| All |  |  |  |  |  |  |
|  | **2009** | 2996 (3.23) | 2120 (70.83) | 741 (24.76) | 93 (3.11) | 39 (1.3) |
|  | **2010** | 3322 (3.47) | 2195 (66.07) | 998 (30.04) | 97 (2.92) | 32 (0.96) |
|  | **2011** | 3228 (3.22) | 2931 (90.8) | 175 (5.42) | 87 (2.7) | 35 (1.08) |
|  | **2012** | 3466 (3.31) | 3153 (90.97) | 184 (5.31) | 87 (2.51) | 42 (1.21) |
|  | **2013** | 3496 (3.36) | 3123 (89.33) | 196 (5.61) | 94 (2.69) | 83 (2.37) |
|  | **2014** | 3477 (3.33) | 3164 (91) | 165 (4.75) | 94 (2.7) | 54 (1.55) |
|  | **2015** | 3283 (3.13) | 2951 (89.89) | 151 (4.6) | 114 (3.47) | 67 (2.04) |
|  | **2009-2015** | 23268 (3.29) | 19637 (84.41) | 2610 (11.22) | 666 (2.86) | 352 (1.51) |

(1) Principal or additional diagnosis ICD-10-AM code: *O04.5–O04.9 Medical abortion, complete or unspecified* from HMDS. (2) The ICD-10-AM category *O09* *Duration of pregnancy* was used to compute the duration of pregnancy for a specific group of high-risk pregnancies, including abortion: (Refer ACS *1518 Duration of pregnancy* in NCCH 2002). We used the following codes that indicate ranges of completed weeks of pregnancy: *O09.0: <5 weeks; O09.1: 5-13 weeks; O09.2: 14-19 weeks; O09.3: 20-25 weeks; O09.4: 26-33 weeks; O09.5: 34-37 weeks; O09.9: unspecified or not reported.* sup: cell counts less than 10 are suppressed.
