# Supplementary material for: Indirect evidence of sex-selective abortion practices to the imbalanced sex ratio at birth in Australian migrant populations
Source: PLOS Glob Public Health. 2025 May 28;5(5):e0004672. doi: 10.1371/journal.pgph.0004672 (PMC12118887; doi:10.1371/journal.pgph.0004672)
Supplement: S5 Table — (DOCX) [file pgph.0004672.s008.docx]

| **S5 Table. Male-to-female ratios of singleton births in Australia by mother's country of birth and previous history of induced abortion in WA, 1994-2015** | | | | | | | | |
| --- | --- | --- | --- | --- | --- | --- | --- | --- |
|  |  |  |  |  |  |  | % Of induced abortions by previous sex | |
| **Country** | **n1** | **SRB**^1^ (all WA) | **n2** | **SRB**^2^ | **n3** | **SRB**^3^ | **Any previous female** | **Any previous male** |
| Australia | 415,291 | 1.05 (1.04,1.06) | 385,692 | 1.05 (1.04,1.06) | 29,599 | 1.07 (1.04,1.09) | 7.62 | 7.64 |
| China | 5,238 | 1.11 (1.05,1.17) | 4,898 | 1.09 (1.03,1.16) | 340 | 1.39 (1.12,1.73) | 7.62 | 7.05 |
| India | 7,917 | 1.06 (1.02,1.11) | 7,014 | 1.06 (1.01,1.11) | 903 | 1.10 (0.96,1.25) | 14.03 | 12.91 |
| New Zealand | 23,079 | 1.04 (1.02,1.07) | 21,376 | 1.04 (1.01,1.07) | 1,703 | 1.05 (0.95,1.15) | 7.94 | 8.74 |
| UK | 52,328 | 1.03 (1.01,1.05) | 49,921 | 1.03 (1.01,1.05) | 2,407 | 1.03 (0.95,1.12) | 5.44 | 5.01 |
| Vietnam | 4,355 | 1.09 (1.02,1.15) | 4,078 | 1.08 (1.02,1.15) | 277 | 1.11 (0.88,1.41) | 6.31 | 7.58 |
| Lebanon | 351 | 1.03 (0.83,1.27) | 340 | 1.01 (0.82,1.17) | 11 | 1.75 (0.51,5.98) * | NA | NA |
| Philippines | 4,139 | 1.10 (1.04,1.17) | 3,987 | 1.10 (1.04,1.17) | 152 | 1.11 (0.81,1.53) | 3.72 | 5.74 |

^1^ SRB for all WA; ^2^ SRB for observations with no history of induced abortion; ^3^SRB for observations with history of induced abortion; * small cell counts (<100) and unstable.
