# Supplementary material for: Indirect evidence of sex-selective abortion practices to the imbalanced sex ratio at birth in Australian migrant populations
Source: PLOS Glob Public Health. 2025 May 28;5(5):e0004672. doi: 10.1371/journal.pgph.0004672 (PMC12118887; doi:10.1371/journal.pgph.0004672)
Supplement: S6 Table — (DOCX) [file pgph.0004672.s009.docx]

**S6 Table. Male-to-female ratios of singleton births in Australia (WA, NSW) by mother's country of birth and stratified by previous sex and birth year, 1994-2015**

| **Country; Previous sex** | | **1994-2009** | **2010-2015** |
| --- | --- | --- | --- |
| **Australia** | Male | 1.049 (1.038,1.059) | 1.060 (1.045,1.075) |
|  | Female | 1.057 (1.047,1.068) | 1.045 (1.030,1.060) |
|  | All | 1.061 (1.057,1.065) | 1.060 (1.053,1.067) |
| **China** | Male | 1.063 (0.998,1.132) | 1.089 (1.021,1.162) |
|  | Female | 1.086 (1.019,1.158) | 1.134 (1.062,1.211) |
|  | All | 1.052 (1.024,1.080) | 1.079 (1.043,1.116) |
| **India** | Male | 1.052 (0.953,1.162) | 1.059 (0.985,1.138) |
|  | Female | 1.165 (1.053,1.289) | 1.134 (1.058,1.216) |
|  | All | 1.024 (0.985,1.064) | 1.040 (1.002,1.080) |
| **New Zealand** | Male | 1.037 (0.979,1.099) | 1.023 (0.946,1.107) |
|  | Female | 1.052 (0.991,1.117) | 1.051 (0.969,1.140) |
|  | All | 1.059 (1.036,1.083) | 1.069 (1.032,1.108) |
| **UK** | Male | 1.04 (0.996,1.085) | 1.075 (1.011,1.144) |
|  | Female | 1.051 (1.005,1.098) | 0.988 (0.928,1.052) |
|  | All | 1.055 (1.036,1.073) | 1.051 (1.019,1.084) |
| **Vietnam** | Male | 1.004 (0.941,1.072) | 1.035 (0.941,1.139) |
|  | Female | 1.092 (1.022,1.167) | 0.970 (0.878,1.073) |
|  | All | 1.045 (1.017,1.074) | 1.055 (1.005,1.109) |
| **Lebanon** | Male | 1.020 (0.942,1.104) | 1.044 (0.918,1.188) |
|  | Female | 1.148 (1.058,1.246) | 1.198 (1.050,1.365) |
|  | All | 1.044 (1.016,1.073) | 1.054 (1.001,1.110) |
| **Philippines** | Male | 1.090 (0.998,1.190) | 1.049 (0.939,1.171) |
|  | Female | 1.076 (0.981,1.180) | 1.181 (1.052,1.327) |
|  | All | 1.096 (1.059,1.135) | 1.129 (1.070,1.190) |
