# Supplementary material for: Indirect evidence of sex-selective abortion practices to the imbalanced sex ratio at birth in Australian migrant populations
Source: PLOS Glob Public Health. 2025 May 28;5(5):e0004672. doi: 10.1371/journal.pgph.0004672 (PMC12118887; doi:10.1371/journal.pgph.0004672)
Supplement: Attachment — Submitted filename: Response to Reviewers_1.pdf [file pgph.0004672.s011.pdf]

Response to Reviewers' Comments

Dear Editor in Chief,

We are pleased to submit a revised version of our manuscript titled "*Indirect evidence of sex-selective abortion practices to the imbalanced sex ratio at birth in Australian migrant populations*" for further consideration as a research article in PLOS Global Public Health.

We thank you, and the reviewers for your interest in our manuscript and the valuable comments. We have accepted all those comments suggested by the reviewers and made significant changes in the respective sections of the manuscript. The detailed point-by-point responses to the reviewers' comments are provided below.

We believe that the current version of the manuscript now meets the requirements for acceptance and look forward to your decision.

Sincerely,

Amanuel Gebremedhin (PhD) corresponding author

EDITOR COMMENTS

Journal Requirements:
1. Please provide an Author Summary. This should appear in your manuscript between the Abstract (if applicable) and the Introduction, and should be 150–200 words long. The aim should be to make your findings accessible to a wide audience that includes both scientists and non-scientists. Sample summaries can be found on our website under Submission Guidelines: https://journals.plos.org/globalpublichealth/s/submission-guidelines#loc-parts-of-a-submission.

Author RESPONSE:

We thank the editor for the suggestion of including the author summary, which we believe will make our paper easily accessible to wider audience including non-scientists. As suggested, we have now included the author summary in the section immediately after abstract (page 3, Lines 41-60).

REVIWER #1 COMMENTS

1. This is an interesting study. I am not a quantitative analyst so I cannot comment on the statistical analysis. However, I have some feedback which should hopefully improve the paper. Most points are fairly minor. The last point is the most substantial but still not onerous. The authors need to explain technical terms, e.g. the stopping rule (among others).

Author RESPONSE:

Thank you for the complement and the suggestion to explain technical terms used in our paper.

As suggested, we have now moved relevant details that was included in our supplementary document to the main body of manuscript. For example, we have included a selection process as well as variable definitions such as parity, time period, stopping rule and infant sex (pages 7-8, line 121-158) in the Methods section as shown below.

"Selection process

The final analytical sample included the country of maternal birth with at least 20,000 births during the study period (eight countries). These were Australia (n= 2,175,252), the UK (n=102,538), New Zealand (n=68,264), China (n=68,003), India (n=48,302), Vietnam (n=41,231), Lebanon (n=35,701), and the Philippines (n=32,652).

Operational definitions

Mother's country of birth: We classified all births based on the birth country of the mother according to the Australian Standard Classification of Countries for Social Statistics (ASCSS) 1990,[16] the Standard Australian Classification of Countries (SACC) 1998,[17] the Standard Australian Classification of Countries 2nd edition (SACC 2nd edition)[18] and the Standard Australian Classification of Countries 2011 (SACC2011).[19] For records from NSW, we mapped the Australian Bureau of Statistics (ABS) 4-digit country of birth codes with the names according to the ABS Standard Classification of Countries. In WA, mothers' country of birth was ascertained using the mother's place of birth variable in the Birth Registration data or country of birth variable from the mother's Hospital Morbidity Data Collection (HMDC) records. The mother's place of birth variable in the Birth Registration is a free text variable including city, province, and country. We extracted the country of birth from the free text by developing a rule-based natural language processing algorithm and merged it with the MNS data. [20, 21]The rule-based approach means that the algorithm is tailored to fit the dataset as opposed to applying machine learning / statistical techniques that are more generalizable but require labelled training data which are resource and time intensive to construct. The country of birth information from the HMDC was used to ascertain the mother's place of birth and to retrieve the missing values in the Birth Registration database (i.e., birth records before 2000). Thus, the mother's country of birth was complete. To ensure enough statistical power for computing sex ratios in the stratified analyses, we included the following eight countries each providing at least 20,000 births over the study period: Australia, China, India, New Zealand, the UK, Vietnam, Lebanon, and the Philippines. This approach has been adopted by other studies. [13, 22]

Infant sex: Defined as the biological sex at birth, categorized as male or female.

*Parity**:** defined as the total number of previous pregnancies, categorized as (0: First birth, no previous birth; 1: Second birth, one previous birth; 2: Third birth, two previous births and 3+: Fourth or higher order births, three or more previous births).*

Time Period: The birth year was categorized into (1994-99, 2000-2004, 2005-09, and 2010-15) periods.

The stopping rule: a fertility decision where families chose to have no more additional children after having a desired sex composition of their children (usually after a son). For other detailed operational definitions included in the supplementary file (S1 file)."

2. If the terminology is gendered in the data (i.e. "mother" rather than "pregnant person" then it is worth stating this.

   Author RESPONSE: As suggested, we have included a sentence that clarifies the terminology used in the data source. We have included the following text in the methods section on the 'operational definition' subs section; page 7-8, lines 144-146.

   "*In this study, the term 'mother' is used as it reflects the terminology in the original data source, which records information on the birthing parent as 'mother of the baby.'"*

3. Just because someone is born outside Australia does not mean they are not an Australian citizen. If you want to use Australian-born v. Chinese-born as a proxy for those with Chinese heritage, you would need to note that those who are Australian-born could still have Chinese heritage. If the data

includes the mothers' stated ethnicity then it would be best to use that. Otherwise, you would need to note the limitations of using these proxies.

Author RESPONSE: Thank you for your comment. We acknowledge that being born outside Australia does not necessarily mean an individual is not an Australian citizen and that Australian-born individuals may still have Chinese heritage for example. Unfortunately, the dataset does not include comprehensive ethnicity information, rather aboriginal status information, which is less relevant to this analysis, Therefore, we used maternal country of birth as a proxy for cultural background.

We have now added a statement in the Limitations section to clarify this on page 19, line 405-406:

"We also acknowledge that using maternal country of birth as a proxy may not fully capture the nuances of cultural and ethnic background…."

4. It is important to note that just because the authors do not observe a skewed sex ratio among certain ethnic groups does not mean that sex selection is not occurring. i.e. It may be that a roughly equal number of parents are selecting for males as for females, hence it does not result in a skew. However, such sex selection, even if it does not result in a skew, is still problematic. For example, some prospective parents (a sizable number of whom are white) travel overseas and incur great expense to use IVF and PGD in order to select the sex of their embryos. If this occurs among white people pre-pregnancy it is plausible that it would be occurring post-pregnancy as well.

Author RESPONSE: We thank the reviewer for this feedback. We acknowledge that the absence of skewed sex ratios among certain ethnic group does not rule out individual instances of sex selection. It is possible that an equal number of parents are selecting for males and females, resulting in no overall imbalance in SRB. However, previous evidence suggests that pre-pregnancy sex selection via IVF and PGD has a minimal impact on overall SRB. In Australia, ART usage is relatively low compared to other high-income countries [PMID: 28069012], and births conceived through these methods generally follow natural sex ratio patterns. While some individuals may pursue ART or travel overseas for sex selection, this is unlikely to significantly influence population-level trends.

Moreover, our study focuses on broader SRB patterns as an indirect indicator of sex selection at the population level and does not capture individual-level decisions that may not result in skewed ratios. To clarify this, we have added the following statement to the Discussion section: page 18, line 367-370.

" While our study examines population-level SRB, individual instances of sex-selection may still occur without distorting overall result. Pre-pregnancy selection via assisted reproduction, though possible, the overall influence on population-level SRB is relatively small."

5. Explain why you have not used more recent data.

Author RESPONSE: We appreciate for the request to explain why recent data were not used**.** The study period (1994–2015) was selected based on the availability and completeness of linked perinatal data in both jurisdiction at the time of analysis. Data linkage processes (governance) require significant time for validation and quality assurance, which can result in a lag in the availability of more recent data (at least in Australian data linkage case), more importantly for sensitive data that includes perinatal information such as abortion history, date of birth.

However, using a longer historical period allows us to examine trends over time and capture potential shifts in SRB patterns among different migrant populations. While more recent data would be valuable, the comprehensive nature of our dataset, covering over two million births across two major Australian states, provides robust insights into the research question.

6. Explanation of line 235 for why abortion after 20 weeks cannot explain SRB, and line 238, needs to be clearer.

   Author RESPONSE: We appreciate the reviewer's feedback regarding the clarity of our explanation. We acknowledge that the statement may have been ambiguous and have revised it for greater clarity. Specifically, we recognise that the explanation in lines 235 and 238 regarding why abortion after 20 weeks cannot explain the male-biased SRB required further clarification.

   To clarify, in WA, medically induced abortions after 20 weeks of gestation that result in either a stillbirth or a live birth (in rare cases) are recorded in the Midwifery Notification System (MNS), a perinatal database that includes newborn sex. Since our SRB estimates are based on recorded births in this system, any sex differences resulting from abortions after 20 weeks would be captured in our data. Therefore, the observed male-biased SRB cannot be attributed to terminations occurring beyond 20 weeks. Additionally, due to the lack of reliable data on how abortion laws are implemented across jurisdictions, it remains unclear whether sex-selective procedures occur within the state, interstate, or overseas. However, if sex-selective abortion is practiced within state of residence (e.g in WA), it would likely occur earlier in pregnancy when such procedures are more accessible and legally permissible.

   To improve clarity, we have revised the relevant section in the manuscript as follows on page 18 lines 357-370.

   "Abortion laws in Australia are generally decriminalised with varying restrictions on gestation and consent requirements (S2 Fig). For example, in WA, abortion was permitted up to 20 weeks, requiring approval from two doctors beyond this limit. As of March 2024, the new WA abortion reform reduced this requirement to one doctor's approval and gestational limit of 23 weeks. However, abortion after 20 weeks cannot explain the male–biased SRB in our study, as these cases would have been recorded in the MNS data with their sex information and included in our SRB estimates. The lack of reliable data on how abortion laws are implemented across jurisdictions makes it unclear whether sex-selective procedures occur within the state, interstate, or overseas. However, if sex-selective abortion does occur within the state of residence, it is more likely to happen earlier in pregnancy when such procedures are more accessible and less restrictive legal environments."

7. There are more harms than those listed on lines 247-248 (i.e. reinforcing gender essentialism and gender stereotypes).

   Author RESPONSE: Thank you for your valuable feedback. We acknowledge that the original text did not fully capture the broader harms associated with imbalanced sex ratios, particularly the reinforcement of gender essentialism and stereotypes.

   To address this, we have revised the sentence to explicitly include these consequences, along with other societal harms on the last paragraph of the discussion section, page 18, line:377-384:

   " Consequential demographic imbalances can lead to marital squeeze, [1] reinforce gender essentialism and stereotypes, and contribute to broader societal harms, including increased violence, restrictions on women's reproductive autonomy, and negative mental health impacts. [1] These imbalances also undermine gender equality by reinforcing discriminatory norms and limiting women's rights. This underscores the importance of

ongoing SRB monitoring, enforcement of existing guidelines, and public awareness campaigns on the consequences of sex selection.".

8. The point made in the last line is quite important as a way of preventing sex selective abortion, but some argument for it needs to be made earlier in the piece, not just mentioned at the very end. E.g. The fact that parents can learn the sex of their fetus, have an abortion in the first trimester and do not have to give a reason for the abortion (and it is impractical to attempt to do so anyhow) means that the only feasible way to prevent sex selective abortion is to stop including this information in NIPT results. You may wish to reference Browne's paper "Why parents should not be told the sex of their fetus" to support this point.

   Author RESPONSE: Thank you for your insightful comment. We agree that the argument for restricting the disclosure of fetal sex in NIPT results as a means to prevent sex-selective abortion should be introduced earlier in the manuscript rather than mentioned only at the conclusion section. We appreciate this suggestion, as it strengthens the coherence of our argument, and we believe these revisions improve the clarity and impact of our findings and recommendations.

   To address this, we have made the following revisions:

   Introduction: as suggested, we now introduce ethical concerns surrounding NIPT and its potential role in facilitating sex-selective abortion. We highlight that, unlike ultrasound, NIPT enables fetal sex determination as early as 10 weeks, providing parents with more time to seek termination in jurisdictions where abortion laws do not require disclosure of reason. We have also incorporated Browne's (2017) argument that restricting fetal sex disclosure is a feasible approach to preventing sex-selective abortion.

   Discussion: We have expanded the discussion to emphasize that as NIPT becomes more widely available and affordable, pressure may mount to relax existing guidelines prohibiting its use for non-medical sex selection. Given the risks associated with sex-selective abortion, we argue that restricting the disclosure of fetal sex remains one of the most practical and ethical solutions.

   To reflect these points, we have revised the Introduction and Discussion sections as follows**:**

   Introduction; page 4 -5 lines 76-90

   "*The increasing availability of non-invasive prenatal testing (NIPT), which can determine fetal sex as early as 10 weeks, has raised some ethical concerns regarding its potential misuse for sex-selective abortion. Unlike ultrasound, which detects fetal sex later in pregnancy, NIPT allows for earlier opportunities for sex selection, particularly in settings where abortion laws do not require a reason for termination. While there is no direct evidence linking NIPT to increased sex-selective abortion, [6] concerns remain that its accessibility could contribute to such practices, particularly among communities with a strong son preference. However, studies indicate an increase in male-biased SRB among Asian migrants, in high-income countries such as the US,[7, 8] Canada,[9, 10] UK,[11] and Australia.[12, 13] Restricting the disclosure of fetal sex in NIPT results has been proposed as a feasible approach to mitigating sex-selective abortion.[14] Given projections of growing sex imbalances at birth due to pre-natal sex-selection practices.[2] It is crucial to examine how these practices may influence SRB trends, particularly in multicultural settings like Australia, where immigrants from countries with historically skewed SRB may maintain son preference and practice prenatal sex selection in their new countries of settlement."*

   Discussion: page 17 lines 334-342

   "*The use of sex–determining technologies, such as ultrasound has led to an increase in sex–selective abortion in Asian countries,[5] and it is feasible that the ease, timing, and accuracy of NIPT could further facilitate this*

practice.[6] Unlike ultrasound, NIPT for determining sex is available much earlier in pregnancy, at 10 weeks of gestation, giving families ample time to seek termination services (considering the legal gestational age for termination of pregnancy in each jurisdiction). In Australia, where abortion laws do not require parents to disclose their reason for termination, restricting the disclosure of fetal sex through NIPT has been suggested as one of the most effective ways to prevent sex-selective abortion.[14]"

Reviewer #2:

1. The analysis using an intercept-only logistic regression model to estimate sex ratios (M/F ratios) at birth and their 95% confidence intervals (CIs) is simple and easy to interpret. However, the data is basically population drawn and the overall presentation is descriptive, rather than inferential. Therefore, statements such as ,"Our findings strongly support the hypothesis of the practice of aborting females in Australia by immigrants from countries with a strong son preference, such as India and China." , may be a bit strong in itself.

Author RESPONSE:

We sincerely appreciate the reviewer's feedback and recognise the concern regarding the strength of our conclusions. We acknowledge that our study primarily provides observational and descriptive evidence rather than inferential analysis. In response to this concern, we have revised the wording in the Discussion and Conclusion sections to reflect a more cautious interpretation of our findings.

That said, we believe our study rather has a comparative advantage over previous research in several key aspects mentioned below.

- Unique Population-based cohort with comprehensive data: Our study is based on a large, population-based cohort from two major Australian states with nearly complete (>99%) birth registrations. Remarkably, our population based linked data includes sex assigned at birth, a variable that is not available in most demographic databases in Australia and likely many other countries. This comprehensive data enabled us to compute conditional sex ratios at birth (SRB), defined as number of males born per 100 female depending on previous birth sex allowing for an indirect estimation of imbalanced SRB potentially linked to prenatal sex determination and sex-selective abortion.
- Beyond Intercept-Only Odds Ratios: While the intercept-only logistic regression model provides a straightforward way to estimate SRB, we have complemented these findings with additional sensitivity analyses:
  - SRB stratified by induced abortion history from hospital morbidity data in WA.
  - Examination of the role of stopping rules using the sex-specific Parity Stopping Ratio (PSR), which provides further insight into sex-based fertility decision-making.
  - Trend analysis of SRB over time to examine temporal shifts for each country included in the study.
- Linked Hospitalization data on induced abortion (WA sub cohort): To provide further context and validation of our results, we also examined hospitalisation records for induced abortions, stratified by gestational age and admission year. This complementary dataset provides additional indirect evidence that aligns with the patterns observed in our perinatal registry data.

To reflect these, we have revised statements in the manuscript to as follows:

Strengths and Limitations section:
Page 20: Line 404-408

"It is important to acknowledge that our study could not determine the specific contribution of pre-pregnancy sex selection through assisted reproduction or the direct impact of NIPT in our findings. Further research such as prospective cohort studies leveraging linked health data is warranted to establish causal pathways."

Conclusions sections: page 20: lines 411-414

“*Our findings suggest patterns consistent with male biased SRB among certain immigrant groups in Australia, particularly from countries with a strong son preference, such as India and China. While this may be indicative of prenatal sex-selection, our study does not establish causality.”*

2. The authors use the term, ‘parity’ which most people would interpret as ‘equivalence’. The investigators do not really define, ‘parity’ up front for the reader in this context. For example, tell the reader what a ‘parity 0-1-2’ is or refer them to the appropriate appendix early on. Only after reading through the document does this become clear.

Author RESPONSE: We appreciate this observation and have clarified the definition of parity earlier in the Materials and Methods section. Specifically:

Original statement: *"Parity: defined as the total number of previous pregnancies, categorized as (0: no previous birth; 1: one previous birth; 2: two previous births and 3+: three or more previous births)."*

Revised statement (earlier in the Methods section); page 8, line 152-154

Parity: *defined as the total number of previous pregnancies, categorized as (0: First birth, no previous birth; 1: Second birth, one previous birth; 2: Third birth, two previous births and 3+: Fourth or higher order births, three or more previous births).*

3. The presentation of the Figures 1,2 and 3 could be presented more clearly and interpreted more readily for the non -statistical reader. Why in these figures are there some comparatively wide confidence intervals? Is this due to the sample size or some heterogeneity factors that are not clear?

Author RESPONSE: We appreciate the reviewer’s comment regarding the wider confidence intervals (CIs) in Figures/presented results and have taken steps to enhance clarity using lay language.

As clearly pointed out by the reviewer, the wider CIs, particularly at higher parity (3+), are primarily due to: (i) Smaller sample sizes – fewer births at higher parities naturally result in greater statistical uncertainty, widening CIs; (ii) Heterogeneity in fertility behaviour – In countries with strong son preference (e.g., India), families may continue childbearing until they have a son (stopping rule), leading to greater variability in SRB estimates; and perhaps (iii) Male-biased SRB at higher parity aligns with existing literature on sex selection, though wider CIs at this level require cautious interpretation.

Despite wider CIs at parity 3+, the overall trend of male-biased SRB remains consistent across different analytic approaches we examined including in our sensitivity analysis. Additionally, many estimates still fall outside the expected natural SRB range, supporting the robustness of our findings, which is now clarified this in the manuscript to improve interpretation. Hence, we added explanations in the discussion section discussing the variability in confidence intervals and interpretation in lay language. Specifically, we now included the following statement in discussion section: **page:16; line 309-317.** As suggested, we also revisited our figures for clarity and included a self-explanatory footnote (see Figures 1, 2 and 3).

" Although our findings suggest a consistent trend of male-biased SRB, we note that wider confidence intervals at higher order births (parity 3+) for some countries (e.g., China, India, and Vietnam) reflect smaller sample sizes and greater

variability in fertility behaviours such as stopping rules. Despite this, the overall trend of male-biased SRB remains consistent and aligns with existing literature on sex ratio imbalances among immigrant groups, particularly from countries with a strong son preference. These findings should be interpreted with caution, particularly for higher birth orders where statistical uncertainty is greater. However, most estimates remain outside the expected natural SRB range, reinforcing the validity of our results."

4. The sensitivity analysis explanation on page 10 is not well explained. The investigators state that, according to their sensitivity analyses, the impact of the stopping rule was more notable in Chinese mothers than in Indian (S2 Table). It is not that notable without some further explanation. As in previous parts of the manuscript, use the data from S2 to demonstrate for the reader why that is the case. The S1 file does well to explain the sensitivity procedure. However, as noted, data from the actual S2 table would help in this case.

   Author RESPONSE: We acknowledge the need for more explicit support of our sensitivity analysis findings with data from S2–S6 Tables, including S2 Table as highlighted by the reviewer. To address this, we have revised the Results section to incorporate specific numerical findings from these tables, reinforcing the robustness of our main results while clearly outlining the procedures and operational definitions. For this, we have opted to move few texts from the S1 file to the main result. Additionally, we have expanded the S1 file to provide further explanations of the procedures, operational definitions, and potential interpretations of the sensitivity analysis. Where relevant, we have defined key terms within the manuscript, while more detailed descriptions remain available in the supplementary file (S1 file).

   Revised text in the Results section: page **13-15; line 251-283**

   " According to our sensitivity analysis, the overall Sex Ratio at Last Birth (SRLB, the sex ratio of the final birth when women have ceased their reproduction) was higher than SRB for births to mothers from China, Vietnam, Lebanon, and the Philippines indicating that the imbalanced SRB can be partly explained by stopping rule. Similarly, the PSR (proportion of women with a given number of children who cease childbearing at that specific parity) of male last births was higher than female last births across all parities for births from these countries, but most notably for births to mothers from China. For example, at parity 2 (third birth), 84.9% of mothers from China stopped childbearing after a male birth, compared to 74.4% after female birth. Consequently, the male: female PSR ratio for mothers from China was consistently above 1 at each parity, and 1.09 for all parities combined which implies that that mothers from Chinese origin are 9% more likely to stop childbearing if their last child was a son compared to a daughter. Conversely, the impact of the stopping rule - the tendency of families to continue having children until they have a son - was less evident for mothers from India as only 78.6% of them stopped childbearing following male birth as compared to 75.7% following female births at parity 2 with an overall male: female PSR ratio of 0.96, indicating that they were 4% less likely to stop childbearing if their last birth was male as compared to females. Due to the smaller sample sizes, the PSR estimates for mothers born in other countries were inconsistent across parities (S2 Table). Detailed operational definitions and interpretations used in this analysis is included in the supplementary file (S1 file).

   The result of our sensitivity analysis to the SRB by the sex composition without considering parity order was consistent with the main analyses supporting the hypothesis that the SRB is strongly influenced by the previous birth sex (S3 Table). During the NIPT available period (2009–2015), Indian–born mothers in WA had a higher rate of hospital induced abortions (6.7%) compared to Australian–born mothers (3.6%) with a greater proportion of early term abortions (before 13 weeks of gestation), 93% vs 84% respectively. Notably, the proportion of early abortions among Indian–born mothers increased from 64.3% in 2010 to 97.7% in 2015 (S4 Table). Our sensitivity analyses also suggested an elevated SRB for births from Indian (1.10, 95% CI 0.96–1.25) and Chinese–born (1.39, 95% CI 1.12–1.73) mothers with at least one prior abortion. Additionally, the proportion of induced abortions for Indian and Chinese–born mothers was higher if they previously had at least one daughter as compared with a previous son (S5 Table). In the year between 2010 and 2015, the SRB for births to mothers from

China, India, Lebanon and the Philippines ranged from 1.06 to 1.13 and was higher in comparison to previous years prior to availability of NIPT (S6 Table)."

5. The investigators made a good effort to put all this together assuming the sampling was done properly. The clarity of the statistical explanation of the results could be enhanced as noted in the examples above.

   Author RESPONSE: We sincerely appreciate the reviewer's positive feedback on our efforts in synthesizing the analysis. We acknowledge the need for greater clarity in the statistical explanation of the results and have revised the manuscript accordingly as described above (see response 1 for Reviewer #1 and responses 1, 2 and 4 above for reviewer #2). Specifically, we have: (i). Enhanced the clarity of statistical explanations in the Results section, ensuring that key findings are presented in a more accessible manner; (ii) Revised the discussion to ensure that key results are interpreted in a way that is accessible to a broader audience; (iii) As mentioned above (#4) ,we have Added a supplementary note in the S1 file providing further details on the operational definitions, procedures, model assumptions and sensitivity analyses for interested readers; and (iv) Added clarifications where needed to ensure that statistical methodologies and findings are clearly conveyed without unnecessary complexity.
   For example, the following statement was included in the statistical analysis section on **page 8-9, line 167-176.**

   "*For the parity 0-1 cohort, we presented the sex ratio for each group (parity 0: firstborn; parity 1: second born; M: second birth, firstborn was male; F: second birth, firstborn was female). Similarly, for the parity 0-1-2 cohort, we presented the sex ratio for the following groups (parity 2: third born: no conditioning; MM: third birth, first and second birth were male; FF: Third birth, first and second births were female; Mixed: one male, one female in any order). Small cell observations were suppressed in accordance with data privacy regulations for WA Data Linkage Branch."*

   And sensitivity analysis section. **Page 9, line 177-196**

   "*We conducted several sensitivity analyses to ascertain the robustness of our main findings. Firstly, we assessed the impact of the stopping rule, which reflects families' decision to stop having children after achieving a desired sex composition, mostly son. To assess how this influences fertility decisions and affects sex ratio of the last birth (SRLB)—as the sex ratio of the final birth when women have completed childbearing —we computed the sex-specific Parity Stopping Ratio (PSR). The sex-specific PSR represents the proportion of mothers with a given number of children who cease childbearing after having a male or female child at a specific parity. If the sex of the child does not influence fertility decisions, the sex-specific PSR should be similar, regardless of the sex of the previous birth. We reported these estimates overall and by parity. A higher SRLB compared to the general SRB suggests that the stopping rule has contributed to an imbalance in the sex ratio at birth (S2 Table). Secondly, to maximise the sample size during stratification by parity, we estimated the SRB by the sex of previous births without considering parity order (S3 Table). Finally, to check the potential influence of induced abortion and dispersion of sex-determining technology on SRB, we conducted a separate analysis in a sub-cohort of births in WA reporting the (i) number of hospitalisations with induced abortion stratified by gestational age and separation year; (ii) SRB stratified by the previous history of induced abortion; and (iii) SRB stratified by birth year (S4-S6 Table). The hospital separation data for induced abortion was obtained from the Hospital Morbidity Data Collection in WA."*

   We believe these revisions improve the readability and accessibility of our manuscript. We appreciate the reviewer's insightful suggestions and have made these changes accordingly.
