# Supplementary material for: Indirect evidence of sex-selective abortion practices to the imbalanced sex ratio at birth in Australian migrant populations
Source: PLOS Glob Public Health. 2025 May 28;5(5):e0004672. doi: 10.1371/journal.pgph.0004672 (PMC12118887; doi:10.1371/journal.pgph.0004672)
Supplement: Attachment — Submitted filename: Response to Reviewers_2.pdf [file pgph.0004672.s012.pdf]

Response to Reviewers' Comments

Dear Editor,

We are pleased to submit a revised version of our manuscript titled "*Indirect evidence of sex-selective abortion practices to the imbalanced sex ratio at birth in Australian migrant populations*" for further consideration as a research article in PLOS Global Public Health.

We thank you, and the reviewers, for your interest in our manuscript and the valuable comments.

Please accept our apology for not including the explanation for not including a recent data in the main manuscript while we justified in response to the reviewer's document. As suggested, we have now included a brief statement on the limitation section of the main text acknowledging the limitation (page 19, Lines 392-396) as follows:

"*Moreover, the study period (1994–2015) was determined by the availability of linked perinatal data at the time of the research. While more recent data would provide additional insights, the extensive nature of our dataset, which includes over two million births across two major Australian jurisdictions, ensures that the findings remain robust and provide valuable insights into the research question."*

We believe that the current version of the manuscript now meets the requirements for acceptance and look forward to your decision.

Sincerely,

Amanuel Gebremedhin (PhD) corresponding author
